# Challenges to sharing sample metadata in computational genomics

**DOI:** 10.3389/fgene.2023.1154198

**Published:** 2023-05-23

**Authors:** Nathan C. Sheffield, Nathan J. LeRoy, Oleksandr Khoroshevskyi

**Affiliations:** ^1^ Center for Public Health Genomics, School of Medicine, University of Virginia, Charlottesville, VA, United States; ^2^ School of Data Science, University of Virginia, Charlottesville, VA, United States; ^3^ Department of Biomedical Engineering, School of Medicine, University of Virginia, Charlottesville, VA, United States; ^4^ Department of Public Health Sciences, School of Medicine, University of Virginia, Charlottesville, VA, United States; ^5^ Department of Biochemistry and Molecular Genetics, School of Medicine, University of Virginia, Charlottesville, VA, United States

**Keywords:** metadata, data sharing and re-use, genomics, sample table, data integration

## 1 Introduction

The genomic data deluge has led to challenges with sharing and integrating genomic data. While substantial effort has been devoted to making genomics data easier to share, one challenge that has received little attention is the related goal of sharing genomic *metadata*, or attributes of biological samples. Genomic metadata is distinct from genomic data in many important ways that affect the optimal way to share it. Here, we outline several challenges specific to sharing metadata associated with genomic data. We argue that sharing genomic metadata is an important and underserved area in genomics, and that addressing this strategically could lead to alternative sharing paradigms with potentialto improve the overall computational genomics ecosystem.

## 2 What is metadata in genomics?

**FIGURE 1:**
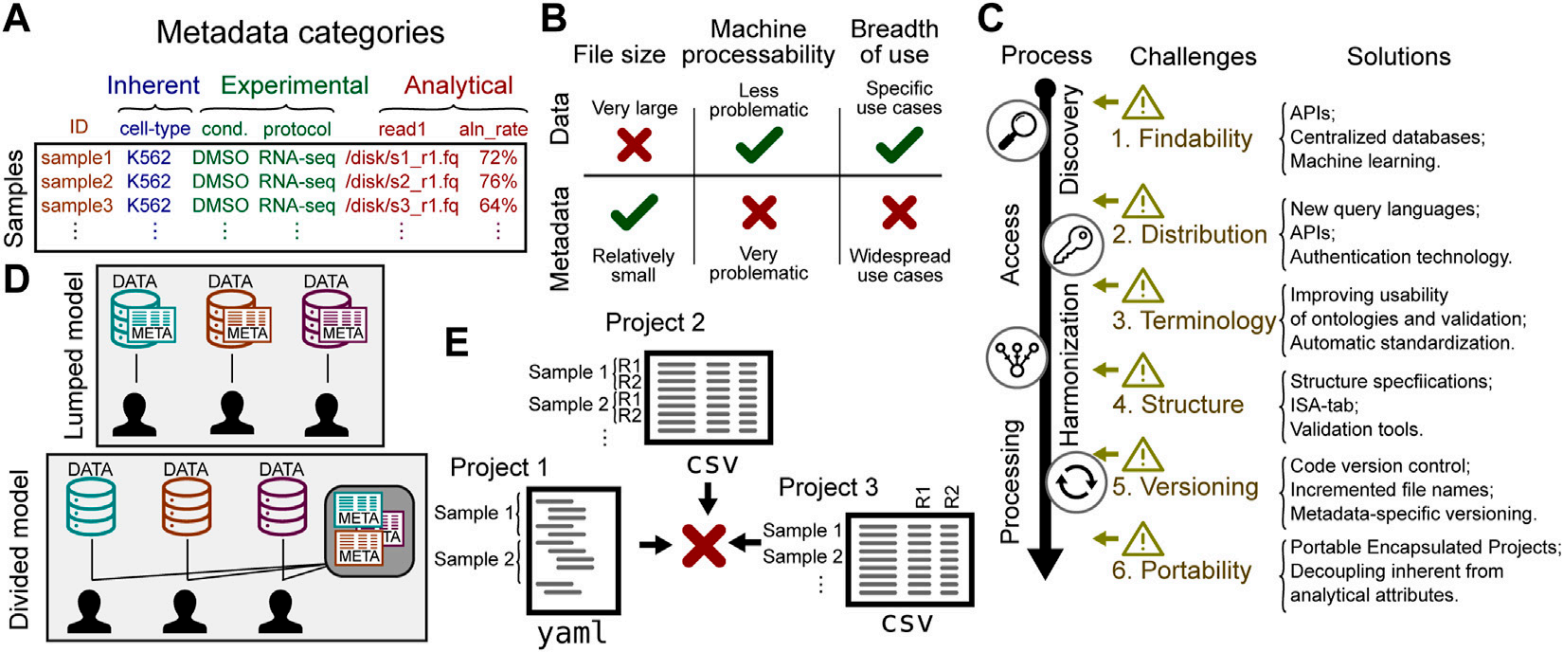
Challenges to sharing genomic metadata. **(A)** Biological sample metadata can be classified by type of attribute. **(B)** Metadata and data have distinct characteristics that affect sharing strategy. **(C)** Challenges in sharing metadata can be found along a process of retrieving data, from discovery to processing. The solutions column offers some recent or future work to address the challenges. **(D)** A lumped model puts data together with metadata; a divided model tailors services to the unique features of each. **(E)** Metadata from different sources may have different structure, making integration difficult.

While much effort has been placed on the idea of sharing genomic data, it is helpful to distinguish between genomic data and metadata. In genomics, data is generally produced by a DNA sequencer, whereas metadata describes the sample from which these sequences were derived. Genomic data is inherently sample-centric: most genomic data is naturally derived from a biological sample. The *attributes* of these samples comprise the metadata. Metadata can be categorized into several types (Figure 1A): 1) inherent attributes describe essential characteristics of a sample, such as species or cell line; 2) experimental attributes describe processing features, such as treatments, experimental conditions, or library preparation protocols; finally, 3) analytical attributes describe inputs or outputs to data analysis, such as parameters or reference genome used. For example, in an RNA-seq experiment, the metadata may include inherent attributes like the sample cell type (K562), experimental attributes like treatment (DMSO), and analytical attributes like paths to data stored in .fastq files.

## 3 Why distinguish metadata from data?

Broad discussions of sharing genomic data do not always distinguish metadata from the data itself. This ignores important differences that affect the challenges and opportunities for sharing. The characteristics of the data and metadata are different enough that the two warrant different strategies for sharing. Three relevant differences with important sharing implications are 1) size; 2) source; and 3) use case. Each of these differences has important implications that change the optimal strategy for sharing (Figure 1B).

### 3.1 Data size

One of the major challenges in sharing genomic data is size. In fact, this is a driving factor that is shaping our sharing strategies and driving huge investments in infrastructure (Schatz et al., 2021; Sheffield et al., 2022). However, metadata is typically much smaller than the data it describes. While genomic data may contain many thousands of sequencing reads for a single sample, the metadata for the same sample might only require a few simple annotations. As such, although it makes sense to avoid data transfer by bringing compute to the data for large genomic datasets (Schatz et al., 2021), this argument simply doesn’t apply to metadata, which is relatively cheap to duplicate and distribute. Lumping the two together therefore creates unnecessary barriers to metadata sharing.

### 3.2 Data source

Another important difference between data and metadata is that metadata is typically created and curated by humans, rather than by machines. Genomic data is overwhelmingly generated by high-throughput sequencers, which have matured to the point of producing standardized file formats which are computer-friendly from the beginning. The primary creator and consumer of these files is machines, as it is impractical for humans to manually explore hundreds of millions of genome fragments. This machine-centric quality creates a self-regulating standardization process for genomic data. In contrast, metadata is more frequently created and consumed by humans. This drastically increases the diversity of metadata, which makes it more challenging to consume, integrate, and analyze by computer. While sequences can all be integrated and processed similarly by software, metadata cannot. This leads to metadata-specific challenges in sharing.

### 3.3 Data use case

Finally, another key sharing-related difference between genomic data and metadata is how it is used. Of course, both data and metadata are likely required for any type of re-analysis, but metadata also has an additional specific use case: it is required for finding the data of interest in the first place. A search for data of interest is likely to need access to metadata in order to determine whether the data is relevant. Finding relevant data requires sifting through lots of potentially irrelevant datasets. As a result, metadata will be much more frequently viewed than data, making it even more important for sharing metadata to be simple, easy, fast, and cheap.

## 4 Challenges to sharing genomic metadata

Given the distinctions between data and metadata, it is clear that sharing metadata warrants a dedicated strategy. This strategy should address challenges specific to sharing metadata, which can be grouped into 6 areas: 1) standardizing terms; 2) standardizing formats; 3) distribution; 4) findability; 5) versioning; and 6) portability. These challenges span the life-cycle of metadata use, including discovery, access, harmonization, and processing (Figure 1C).

### 4.1 Findability

The first step to reusing data is finding it. However, because metadata are not centralized, but scattered across various servers and databases, finding relevant data can be a challenge. In addition to the general challenge of multiple sources to find data, the problem is exacerbated by the inability for computers to parse and index some metadata, such as PDFs or Excel files. Finally, authorization barriers inhibit findability. Though there has been some effort to create centralized search frameworks or open API-oriented systems (Canakoglu et al., 2019), existing tools are still covering only a small amount of the possible search space. Moreover, advances in natural language search indicate an exciting future that could use machine learning models to retrieve relevant research data (Lee et al., 2019).

### 4.2 Distribution

Distribution of genomic metadata is also a challenge. The *status quo* is *ad hoc*; there are a variety of different distribution mechanisms, and none is particularly machine-friendly. Much genomic metadata is deposited onto data-oriented databases, such as GEO or dbGap, where metadata is notoriously difficult to process, leading to a variety of dedicated tools for that purpose (Davis and Meltzer, 2007; Chen et al., 2019; Gumienny, 2019; Choudhary, 2019; Ewels et al., 2020; Cannizzaro et al., 2021; Gálvez-Merchán et al., 2022; Garcia et al., 2022; Khoroshevskyi et al., 2023). Distribution is sometimes intentionally restricted on the basis of privacy. Some patient attributes are protected, requiring authorization barriers, which make it harder to share. Furthermore, even for unrestricted attributes or cell-line data, metadata may be deposited under the same access restrictions as the data itself for convenience, because the repository may not be set up to separate the two (Figure 1D). This convenience violates a tenet of the FAIR philosophy, that metadata should be accessible even if the data itself has restricted access (Wilkinson et al., 2016). To fulfill this could require separating protected from public characteristics for some datasets. Another common distribution mechanism is through attached to journal publications, but this is highly non-standard and is not amenable to easy meta-analysis or reuse. One attempt at making genomic metadata easier to distribute and parse is the GenoMetric Query Language (GMQL), a declarative language that provides abstractions of experimental, biological, and clinical metadata (Masseroli et al., 2015). Modern authentication advances are making it easier to provide granular controlled access. Coupled with advances in API infrastructure, the stage is set for a next-generation of API-based, machine-friendly, data distribution services with granular access provision (Sheffield et al., 2022).

### 4.3 Terminology

A major challenge to sharing and re-using genomic metadata is that terms must match (Xue et al., 2023). One way to address this challenge is with ontologies. Ontologies provide vocabularies with controlled terms and definitions. They may also provide information about relationships among those terms. Creating ontologies is labor-intensive and requires coordination and community, but fortunately, many ontologies already exist for a variety of biomedical use cases (Smith et al., 2007; Hoehndorf et al., 2015; Malladi et al., 2015; Bandrowski et al., 2016). Unfortunately, in practice, researchers do not necessarily use existing ontologies (Fung and Bodenreider, 2019). One barrier is that the benefits of ontologies may become most apparent only in integrative meta-analysis. Therefore, for an individual study, an ontology may be viewed as merely added cost.

We can address this in two ways: either reducing the cost or increasing the value of using an ontology for individual studies. Reducing the cost means lowering the barrier to using controlled terms. There is an opportunity for tools that make it more user-friendly to use an ontology by suggesting controlled terms or mapping existing ontologies while metadata is being created. On the other side, we could approach the problem by adding value to an individual study that uses controlled terms. For instance, we could promote tools that will automatically integrate a new result with external data, even if this is not the primary analysis of the study. One example is gene identifiers: even for a standalone study, researchers want to analyze results in the context of existing public resources, so they must use standardized gene names. Work that develops both standards and annotated datasets for specific data types could encourage others to adopt those standards, such as projects to standardize genomic interval set metadata (Gundersen et al., 2021; Xue et al., 2023). Another possibility is to use machine learning approaches to standardize terminology *post hoc* (Cannizzaro et al., 2021).

### 4.4 Structure

It is not sufficient for two studies to use the same ontology and share controlled terms; they must *also* structure the data in the same way. Genomic metadata frequently adopts a tabular form, with rows corresponding to samples or files, and columns corresponding to attributes of the samples or files. However, genomic metadata may also adopt schema-less, document-based file formats. Furthermore, sample attributes are sometimes encoded in less machine-friendly ways, such as using text formatting or color to mark samples in Microsoft Excel files. Making metadata machine-understandable is a difficult challenge. Even if file formats and general structures are consistent, subtle differences may prevent integration. For example, CSV files can be one row per sample, or one row per file, or one row per sequencing lane (Figure 1E). These distinctions make sample tables non-interoperable, which in turn makes it difficult or impossible to integrate, hindering integrative re-analysis of data. Several attempts have been made to address this issue in general, such as ISA-tab (Sansone et al., 2012), the PEP framework (Sheffield et al., 2021), and META-BASE (Bernasconi et al., 2020). Another way to improve structural interoperability is to improve tooling for validating metadata against schemas. Projects such as JSON-schema (Pezoa et al., 2016), Schema Salad (Crusoe et al., 2022), and LinkML (Moxon et al., 2021) are building required infrastructure, but more work is needed before these become widely used for biomedical research data.

### 4.5 Versioning and identification

Metadata can change. Inherent and experimental attributes are mostly stable but may be edited or added. Furthermore, analytical attributes are frequently added to a metadata table as analysis progresses. Despite clear mutability, metadata tables are often treated as static. Version control is well established for code and has a diverse and multifaceted history for data as well (Klump et al., 2021), but the question of versioning metadata specifically is distinct. A common strategy for versioning sample metadata is to use tools built for code versioning, such as git. However, the fit is not perfect; the line-based nature of git and other code version control systems is less suited to a sample table which may have long lines. A more tailored approach would use a table-cell-based framework, but a bespoke tool for table versioning does not exist. In lieu of this, a common approach is to develop a protocol for recording revisions, typically involving incrementing version numbers in file names (Lawniczak et al., 2022). Also common is to simply not version control metadata. There are clear opportunities for innovation, new standards, and tool development to support the specific needs of metadata versioning.

### 4.6 Portability

A final challenge dealing with sample metadata is its *portability*. By portability, we mean whether relevance is retained if the data or metadata are moved to a different computing environment. Metadata often changes locations. e.g*.*, from one collaborator’s computer to another, from a high-performance computing environment to a web repository to a laptop. In this process, some attributes lose their relevance: Although inherent and experimental attributes tend to be portable, many analytical properties are not. For example, sample tables often include file paths, but paths typically refer to a specific computing environment. Another example is properties used as input to a pipeline. For instance, the reference genome used is often included as an attribute in a table; however, it not a property of a sample itself, but of a particular analysis. If the sample table is reprocessed, this attribute changes. This distinction between portable and non-portable metadata is typically ignored, so genomic metadata includes both in a single table, which renders the table specific to a computing environment and thereby reduces its portability. This problem motivated the Portable Encapsulated Project framework (Sheffield et al., 2021), which allows environment-specific settings to be extracted from the same table into a configuration file that can change with the environment. There is opportunity for new approaches to conceptualize sample attributes in ways to acknowledge this portability problem to treat metadata attributes according to their portability.

## 5 Discussion

Large efforts targeted at standards for genomics data are underway, and helping to improve interoperability of genomic data (Field et al., 2011; Rehm et al., 2021; Velde et al., 2022). Relatively less effort has been focused on metadata specifically; yet genomic metadata differs enough from the data itself to warrant a specific sharing strategy. Metadata-specific challenges include findability, distribution, terminology, structure, identification, and portability; perhaps the greatest challenge to sharing metadata is caused by the overwhelming complexity introduced by its human-curated nature. Addressing these challenges will be critical to improve the interoperability of sample metadata—and interoperability, in turn, is a driver for integration and re-use. Only by solving this challenge will we be able to benefit from the knowledge that emerges from large-scale, systematic data integration. Of course, metadata sharing is just the beginning; important challenges remain with sharing the data itself. Nevertheless, our attempts to integrate data will remain limited until we address the challenges at metadata level that warrant specific attention.
